# Travis County Medical Society

**Published:** 1903-09

**Authors:** 


### Travis County Medical Society.

The Travis County Medical Society held its regular meeting on September 8th. B. M. Worsham, President; M. M. Smith, Secretary. A number of new members were admitted. This takes into the fold about all the physicians of the county.

Dr. Smith, who has recently visited and gone through many consumptives' hospitals in Colorado and New Mexico, entertained the society with a paper descriptive of what he saw and the methods and management of those establishments. Tents are largely used, practically the out of doors treatment, and with good results. Very little drugging is done; that is about played out.

Dr. Ralph Steiner (ear, nose and throat) related several cases of removal of foreign bodies from the trachea.

Dr. Worsham, President, and Superintendent of the State Insane Asylum, was asked to tell about the treatment of consumptives amongst the insane people in his charge. He separates them, of course, as far as possible, from the other inmates, and altogether if confined to bed (although there is no separate hospital provision made for them, as there should be) and depends upon dietetic and hygienic measures principally, using drugs only in complications and as palliatives. He said that since the inauguration of this plan there had been a large decrease in the mortality.

Dr. Steiner said that some criticisms had been made about specialists going to cases in charge of homeopaths and giving antitoxin in cases of diphtheria, for instance; or about physicians going to labor cases where the attending homeopath can not effect delivery, and delivering the child for him, etc., and he wanted to know the sentiment of the society on the subject. A pretty lively discussion followed by Drs. J. S. Wooten, Smith, Hill, Bennett, Steiner, et al.; but, recognizing the fact that they were up against a tough proposition, further discussion and a decision were deferred to next regular meeting. This was advisable, furthermore, because pending the discussion some members were called out. The matter was referred to a special committee, Dr. T. J. Bennett, chairman, who will report at next meeting the principles that should govern in such cases. The general sentiment seemed to be that the going to a case under circumstances as above stated is in no sense a *consultation*, which is *malum prohibitum*.

---

